# Mapping the research landscape of artificial hearts: identifying hotspots and frontiers through bibliometric analysis

**DOI:** 10.3389/fcvm.2025.1613605

**Published:** 2025-09-30

**Authors:** Yufeng Peng, Kewei Peng, Luyao Li, Fanglei Xiong

**Affiliations:** ^1^ Cardiovascular Medicine Department, Zhenhai Hospital of Traditional Chinese Medicine, Ningbo, China; ^2^ The Rehabilitation Department of the Fourth Hospital of Wuhan, Wuhan, China

**Keywords:** artificial heart, bibliometrics, biomaterials, artificial intelligence, cardiovascular medicine

## Abstract

**Background:**

The rising prevalence of advanced heart failure and the shortage of donor hearts underscore the need for artificial hearts. Technological evolution from pneumatic to magnetically levitated systems has occurred, yet critical challenges persist in hemocompatibility, anticoagulation, and transcutaneous energy transmission systems.

**Methods:**

We conducted a bibliometric analysis of 6,310 publications from the Web of Science Core Collection using CiteSpace, VOSviewer, and Bibliometrix. This study aimed to delineate research trends, collaboration patterns, and technological advancements in the artificial heart field.

**Results:**

Global publications showed a 13.9% average annual growth, with citations surging 260-fold. The USA leads research output, with China demonstrating rapid growth. Research focus has shifted from ventricular assist devices towards artificial intelligence control strategies. Magnetic levitation continuous-flow total artificial hearts and soft TAHs emerge as key technological innovations. Frontier research includes biohybrid systems and transcutaneous wireless power transfer.

**Conclusion:**

Artificial heart research displays a three-stage progression: clinical need-driven, technology integration, and intelligent innovation. Enhanced interdisciplinary collaboration is vital to address persistent challenges in biocompatibility optimization and personalized anticoagulation. This is crucial for transitioning the treatment paradigm from mechanical replacement towards physiologically adaptive, long-term solutions.

## Introduction

The incidence and prevalence of advanced heart failure are continuously increasing globally, while the severe shortage of donor hearts for transplantation underscores the urgent clinical need for alternative therapeutic strategies (1–3). Since the initiation of artificial heart development in the 1950s, the field has undergone a revolutionary evolution from pneumatically driven devices to continuous-flow pump technologies, and from temporary bridging therapies to potential permanent replacement solutions (4–6). The Total Artificial Heart (TAH), serving as a complete biventricular replacement device, delivers physiological pulsatile blood flow of 7–9 L/min, thereby not only creating a window for organ function recovery in patients awaiting transplantation but also offering a vital survival option for end-stage patients ineligible for transplant (7, 8). Currently, third-generation devices, exemplified by the SynCardia TAH, have been implanted in over 1,700 cases worldwide, demonstrating their effectiveness as bridge-to-transplant therapy (9).

Despite continuous technological advancements, artificial hearts still face multiple challenges. Biocompatibility issues are paramount: sublethal damage to erythrocytes precedes overt hemolysis under non-physiological shear stress, accelerating phosphatidylserine exposure that triggers thrombin generation. This cascade exacerbates thrombogenicity beyond free hemoglobin release alone (10, 11), decreased mechanical stability of erythrocytes, and the release of free hemoglobin pose significant risks for thromboembolism and end-organ damage (12). Although materials such as titanium alloys are widely used in artificial heart manufacturing, their suboptimal antibacterial and antithrombogenic properties necessitate long-term anticoagulation therapy for patients, potentially leading to hemorrhagic or infectious complications (13, 14). Furthermore, limitations related to the size of existing TAH devices, power supply methods, and long-term durability continue to impede their progression towards destination therapy applications (15, 16).

In recent years, technological innovation has shown diversifying trends. Soft robotics technology, through biomimetic structural designs such as the silicone injection-molded, achieves contraction patterns more akin to the natural heart, significantly reducing the risk of blood damage (17, 18); valveless continuous-flow TAHs, employing magnetic levitation and hydrodynamic balancing technologies, exhibit excellent biocompatibility and system reliability (19); meanwhile, four-chamber designs like the Realheart® TAH, by mimicking the positive displacement pumping mechanism of the native heart, have demonstrated favorable hemodynamic performance in hybrid cardiovascular simulators (20). Concurrently, the introduction of surface modification techniques and AI-assisted evaluation systems opens new avenues for enhancing device performance (21).

However, the field of artificial heart research is highly interdisciplinary, involving materials science, fluid dynamics, clinical medicine, and artificial intelligence, among other areas. Bibliometrics, as a core tool for constructing scientific knowledge maps, analyzes the research dynamics, knowledge base, and frontier hotspots in the artificial heart field over 15 years through visual mapping. By integrating CiteSpace's burst detection technology, VOSviewer's co-occurrence network analysis module, and Bibliometrix's temporal hotspot tracking function, this approach aims to reveal the discipline's cross-disciplinary characteristics and evolutionary pathways, identify the driving role of key technological nodes, such as resting-state functional connectivity analysis, on research paradigms, and explore the potential impact of cutting-edge directions like biohybrid systems and artificial muscle-driven devices on future heart replacement therapies. By integrating engineering breakthroughs with clinical practice evidence, the goal is to provide theoretical support for the optimized design and personalized application of artificial hearts.

## Methods

### Data resources

**Figure 1:**
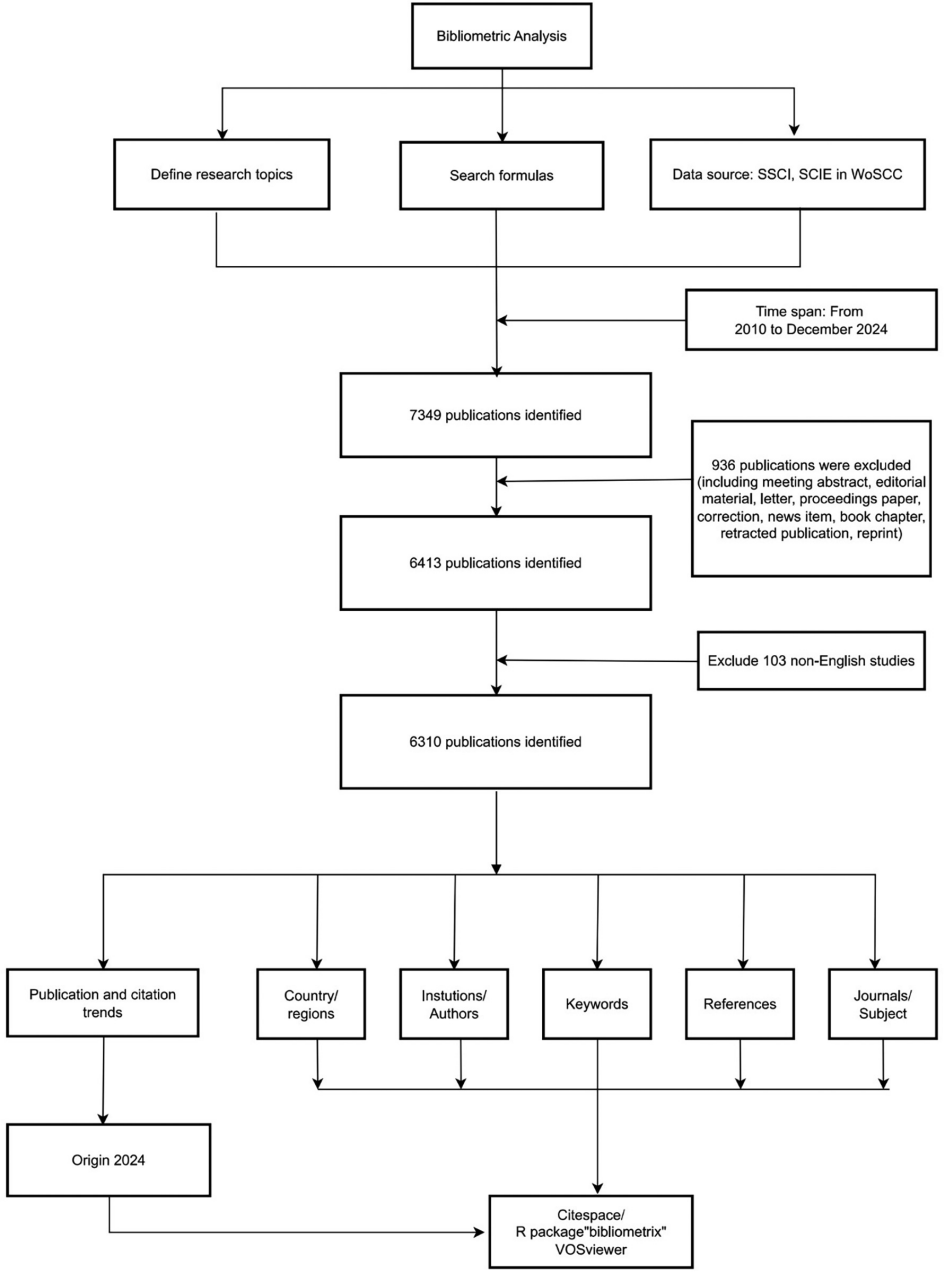
Flowchart of the study.

The Web of Science Core Collection (WoSCC) is globally recognized as an authoritative database and, owing to its comprehensive interdisciplinary coverage, has become the preferred data source for bibliometric research. This database is particularly well-suited for research in highly interdisciplinary fields, such as the study of artificial hearts. Medical Subject Headings (MeSH) is extensively utilized as the controlled vocabulary for indexing and searching biomedical literature within PubMed. To ensure comprehensive retrieval, we adopted a strategy based on MeSH concepts, a widely recognized approach for indexing and retrieving biomedical literature (22). Using the core search concept “artificial heart” within the MeSH framework, we identified three relevant terms, establishing the following search strategy: ([TS = (“Artificial Heart”)] OR TS = (“Artificial Hearts”)) OR TS = (“Hearts, Artificial”)). This study aimed to cover the majority of publications on artificial heart research available in the database to date, specifically including English-language articles published between January 1, 2010, and December 31, 2024. The data retrieved encompassed article titles, abstracts, keywords, author information, country and institutional distributions, citation counts, and details of collaboration networks within relevant fields. Inclusion criteria for document type were restricted to Articles and Review Articles. Excluded were commentaries, editorial materials, meeting abstracts, early access publications, notes, book chapters, letters, retracted publications, and corrections. Finally, through screening of titles and abstracts, 1,039 irrelevant publications were excluded, resulting in the inclusion of 6,310 documents for analysis. The detailed process is illustrated in Figure 1. Detailed searches can be found in Supplementary Digital Content 1.

### Data extraction, cleaning, and standardization

Datasets retrieved from WoSCC included “Full Record and Cited References” and were downloaded in plain text format. The raw data were directly imported into the bibliometric software for analysis, eliminating the need for format conversion. Following the retrieval and download of raw data from WoSCC, titles and abstracts were screened by two independent reviewers (Yufeng Peng and Kewei Peng). Data were exported from the WoSCC database in plain text format, with files individually named following the convention “download_xxx.txt”. The exported data included bibliographic information such as titles, publication years, authors, affiliations, keywords, abstracts, and journal names. Upon completion of the screening process identifying all eligible publications, bibliometric tools—specifically CiteSpace (version 6.4 R1), VOSviewer (version 1.6.2), and Bibliometrix (version 4.1)—were employed to analyze the publication data concerning artificial hearts over the past 15 years. Keyword standardization was performed, involving the consolidation of synonyms, aliases, and the unification of singular and plural forms (23, 24). Data analysis used Processon to draw the flow chart, Origin 2024 to draw the trend chart, SCImago Graphica 1.0.48 to draw the national cooperation visualization chart, VOSviewer 1.6.2 to draw the institutional cooperation visualization chart. Bibliometrix 4.1.3 draws the author diagram, and CiteSpace 6.4R1 draws the keyword outbreak, journal double graph superposition and reference visual analysis diagram.

## Results

### Annual publications, citations, and trends

**Figure 2:**
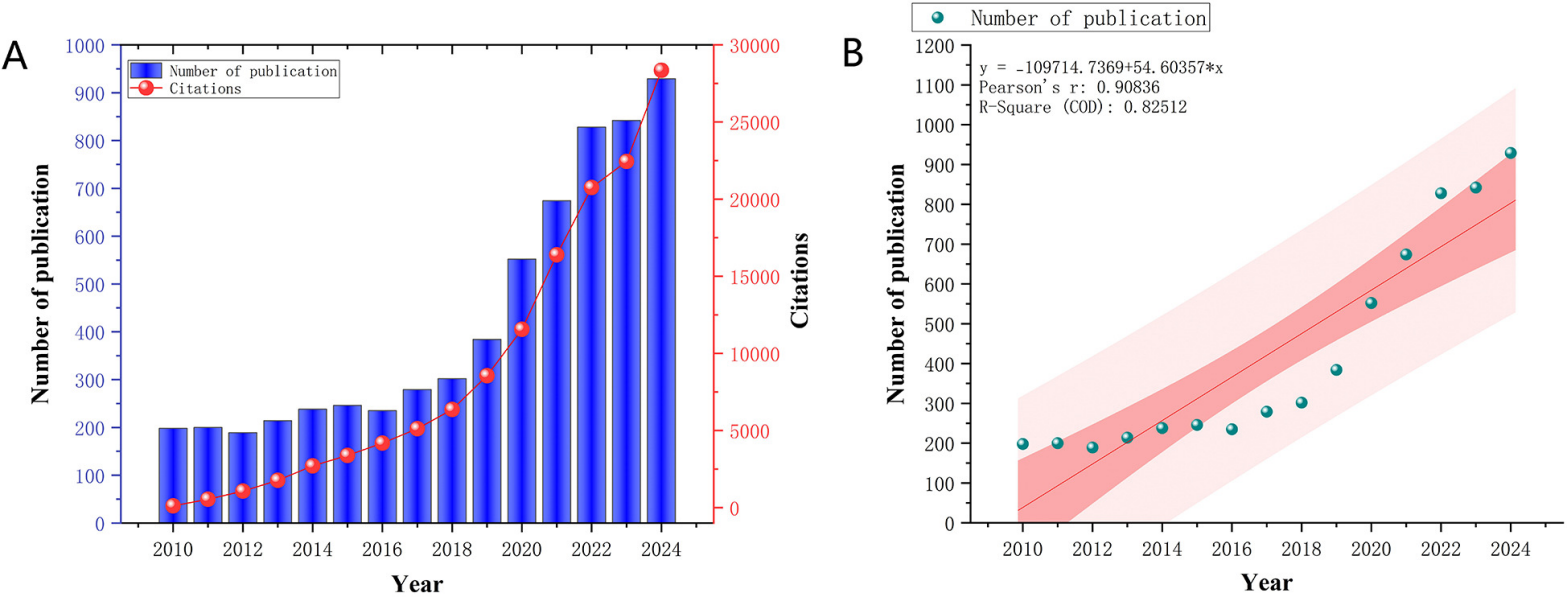
**(A)** Trends in annual publications and cited articles from 2010 to 2024. **(B)** Predicted trends in annual publications and publications from 2010 to 2024.

In this study, a total of 6,310 publications were retrieved. Figure 2A illustrates the annual and cumulative publication trends in the artificial heart field from 2005 to 2024. The period between 2010 and 2024 witnessed significant development in this field, with publication volume steadily increasing from 198 articles in 2010 to 929 in 2024. A particularly rapid growth phase emerged after 2020, contributing to an overall average annual growth rate of 13.9% for the 2010–2024 period. Citation volume experienced even more dramatic growth, soaring from 109 citations in 2010 to 28,355 citations in 2024, representing an increase of approximately 260-fold. A high-growth trajectory in citations was especially prominent between 2020 and 2024, maintaining an average annual growth rate of around 25.1%. Figure 2B further highlights the significant and sustained upward trend in the annual publication volume concerning artificial hearts from 2010 to 2024. Linear regression analysis conclusively confirmed this robust growth, yielding a Pearson's *r* value of 0.90836 and an R-Squared of 0.82512. These statistical metrics indicate a strong positive correlation between publication volume and year. This comprehensive data set not only reflects the continuous expansion in the quantity of artificial heart research but also signifies a concomitant enhancement in research quality and influence. This trend underscores the field's emergence as a prominent research hotspot within medical engineering, with its practical value and clinical application potential gaining widespread recognition across both academia and industry.

### Country/region and institutional analyses

**Figure 3:**
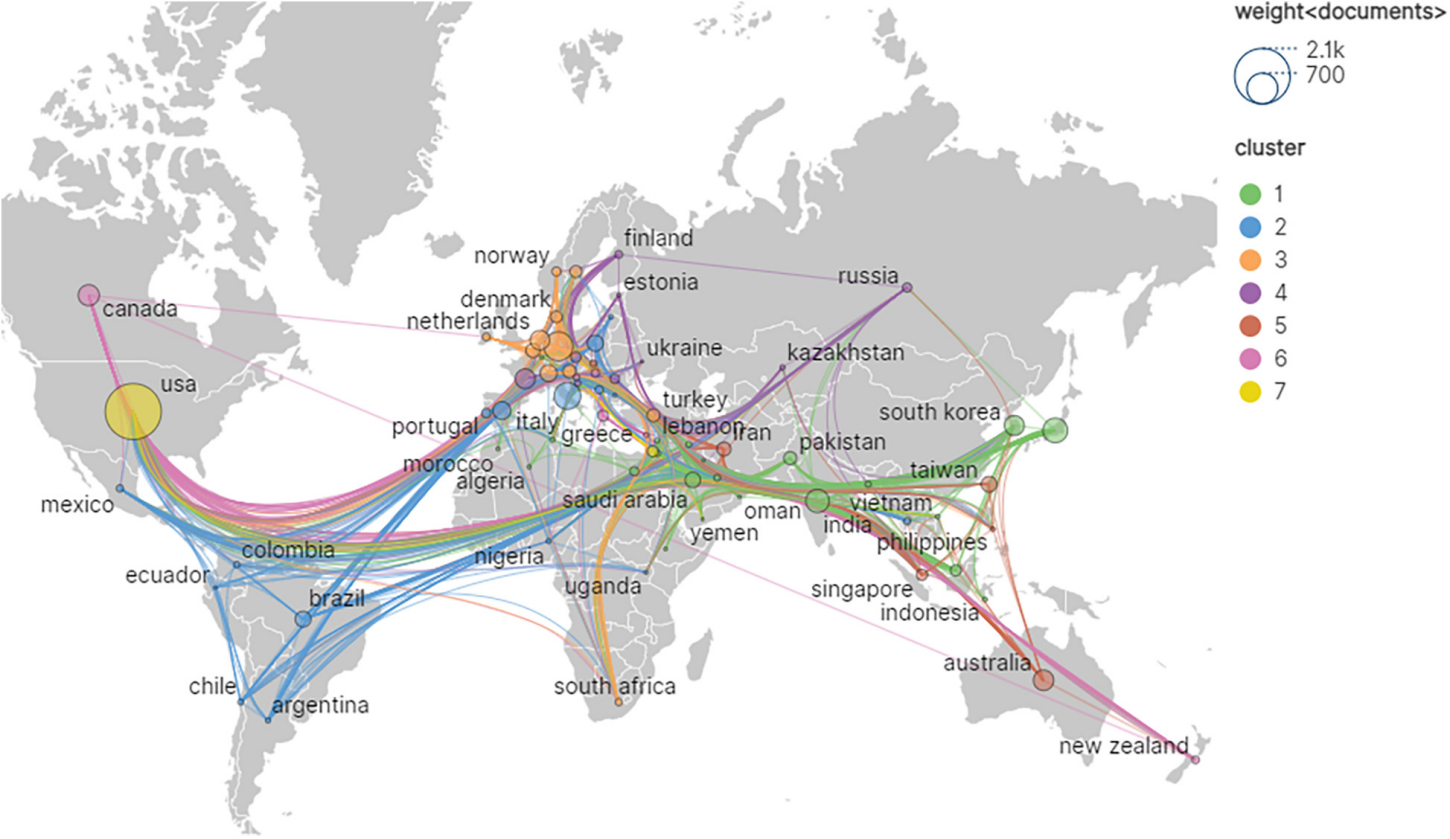
Geographic visualization of country/area collaboration.

Among the retrieved literature, a total of 4,031 institutions from 106 different countries/regions participated in the field of artificial heart research. As presented in Table 1; Figure 3, the global research landscape for artificial hearts exhibits a significant imbalance. The USA unequivocally leads in global research output, with an outstanding 1,449 publications and 40,719 total citations, demonstrating its dominant position in both quantity and influence. Its high average citations per article (28.10) further substantiates the high quality of its research. Although the betweenness centrality (BC) for the USA is 0.03, a relatively modest numerical value, considering its immense output and extensive international collaborations, this may imply that the USA acts as an initiator or participant in numerous bilateral or multilateral collaborations rather than being a singular “bridge” node. Its robust research volume inherently attracts global partnerships, thus forming a highly connected yet moderately centralized network. China ranks second with 925 publications and 10,427 total citations; however, its BC is 0. This indicates that despite China's strong growth momentum and substantial output in research, it has not yet assumed a core “bridge” role within the global scientific collaboration network, meaning it less frequently serves as a connection point between different international collaborative clusters. This may suggest that China's research in artificial hearts is more focused on internal circulation or close collaboration with specific partners. For China to enhance its global influence in the future, it is crucial to actively build broader and more diversified international collaborative networks, transitioning from a high-output nation to a highly connected and influential research hub. Developed European and Asian countries such as Germany, Japan, and Italy, despite significantly lower publication volumes compared to the USA and China, exhibit a BC of 0.03, on par with the USA. Notably, Italy's average citations per article reach 30.32, surpassing that of the USA. This signifies that these countries hold substantial influence in specific areas or in high-quality research, and can play important liaison roles in international collaboration, with their research findings potentially gaining higher recognition and broader dissemination. India and South Korea both have a centrality of 0 and lower average citations, indicating ample room for improvement in their participation and influence within the global research network. Overall, the field presents an international research landscape dominated by North America, with Asia and Europe making concurrent advancements. As the global demand for artificial heart technology continues to grow, international cooperation will become increasingly vital. High-output countries with lower centrality are expected to enhance their BC by actively engaging in international collaborative projects, hosting international conferences, and attracting international talent, thereby moving from major research producers to strong research nations and core hubs. Concurrently, countries with high average citations and high BC will continue to lead the research direction and breakthroughs in this field.

**Table 1:** Publications and citations in the top ten most productive countries/regions and institutions.

Country	Publications	Citations	Average citations	Centrality	Institutions	Publications	Centrality
USA	1,449	40,719	28.10	0.03	Harvard University	177	0.18
China	925	14,711	15.90	0	University of California System	140	0.11
Germany	298	5,605	18.80	0.06	Mayo Clinic	123	0.06
Japan	291	4,803	16.50	0.02	University of London	106	0.02
Italy	289	6,854	23.70	0.07	University System of Ohio	96	0.09
United Kingdom	263	7,422	28.20	0.12	Pennsylvania Commonwealth System of Higher Education (PCSHE)	90	0.03
India	231	4,353	18.80	0.07	Cleveland Clinic Foundation	77	0.02
Korea	198	4,264	21.50	0.03	Institut National de la Sante et de la Recherche Medicale (Inserm)	77	0.06
France	148	3,107	20.90	0.05	Icahn School of Medicine at Mount Sinai	63	0.2
Australia	140	5,099	36.20	0.05	University of Texas System	63	0.03

**Figure 4:**
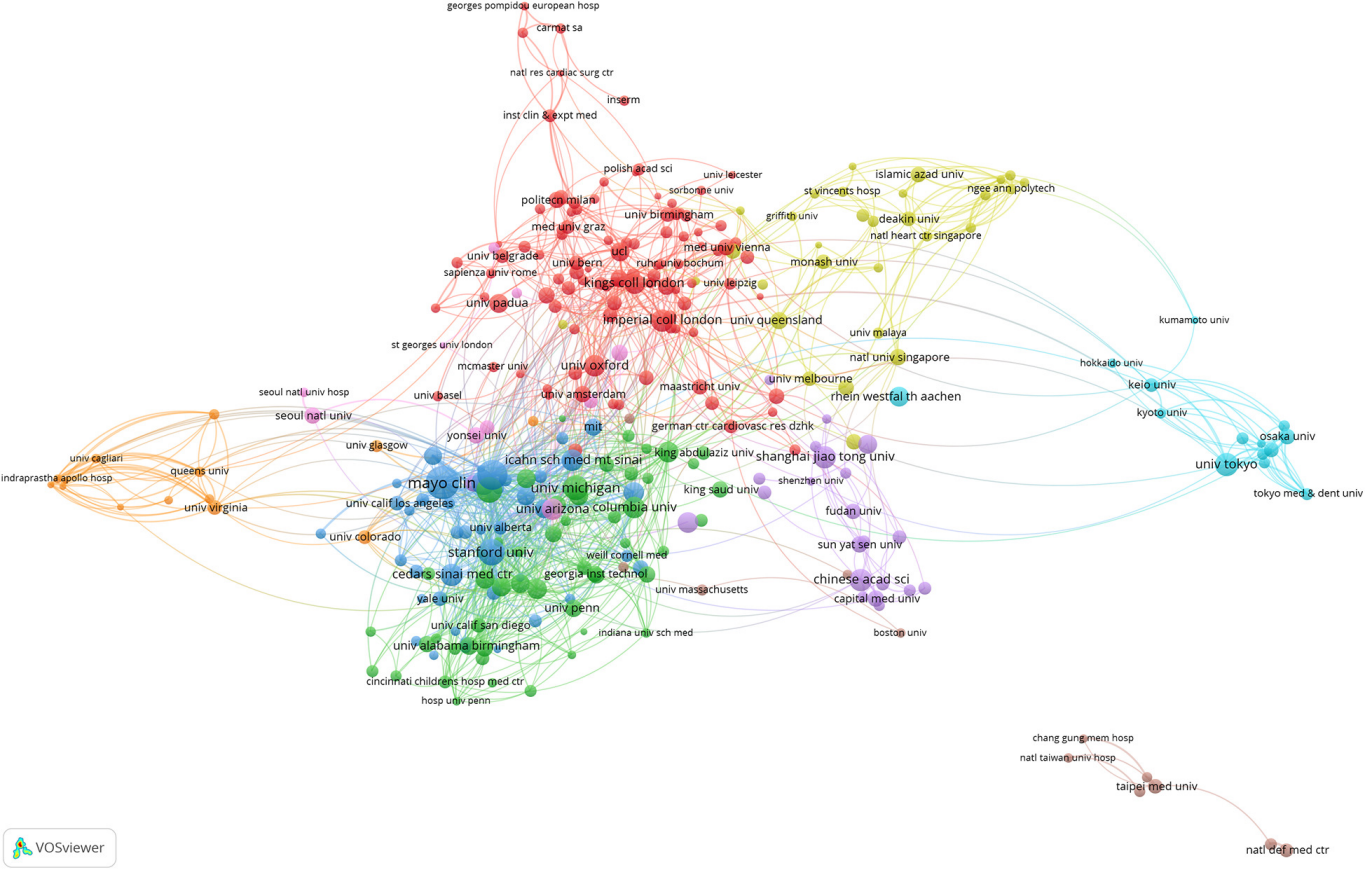
Co-analysis of the top 100 most productive institutions in the network visualization map.

As shown in Figure 4, at the institutional level, Harvard University unequivocally stands as the absolute global leader in artificial heart research, with 177 publications, 9,946 total citations, and an exceptionally high average of 56.20 citations per article. Its remarkable BC of 0.18 is particularly prominent, underscoring that Harvard University not only produces a large volume of high-quality research but also plays a critical “hub” and “bridge” role within the global scientific collaboration network. It connects numerous dispersed research teams and diverse research directions, significantly facilitating knowledge flow and inter-institutional collaboration. Following closely, the University of California System and the Mayo Clinic also demonstrate robust research capabilities. It is noteworthy that while the Mayo Clinic has fewer publications (87 articles) than the University of California System, its average citations per article reached an impressive 64.93, the highest among all institutions, with a BC of 0.06. This indicates that the Mayo Clinic focuses on producing high-quality, high-impact research outcomes and plays a significant role within specific collaborative networks. Other top US institutions, such as the Pennsylvania State University System, the NIH and the University of Texas System, also contribute a substantial amount of research with high average citations, reflecting the generally high quality of their work. However, apart from Harvard University and the Mayo Clinic, the BC of most US institutions is relatively low (0.01–0.04). This may suggest that while internal scientific collaboration within the US is highly active, not all high-output institutions serve as “bridge” functions connecting different large research clusters; rather, they exist as important members within extensive collaborative networks. In contrast, non-US institutions such as Fudan University, INSERM, and Shanghai Jiao Tong University, despite being leading research forces in their respective countries and having considerable publication output, all exhibit a BC of 0. This finding echoes the country-level observations, indicating that these institutions have not yet established an effective “bridge” status within the global institutional collaboration network. This implies they may be more involved in regional or specific bilateral collaborations, rather than serving as connection points between diverse international research groups. The overall data reflects that the institutional distribution in artificial heart research is characterized by US medical schools at its core, with top research institutions from multiple countries participating, which is highly consistent with the field's need to integrate clinical experience and technological innovation.

### Authors and co-cited authors

**Figure 5:**
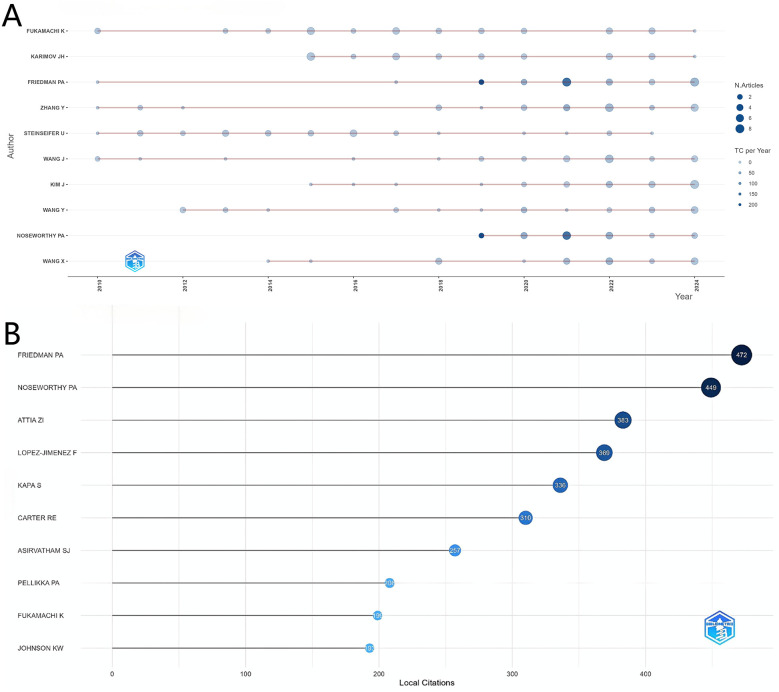
**(A)** Authors’ production over time. **(B)** Most local cited authors.

Analysis of author productivity in the artificial heart research field, depicted in Figure 5A, shows a clear concentration among top contributors. Notably, Friedman PA leads significantly in terms of local citations with 472, establishing him as the most influential core scholar in this domain. Researchers such as Noseworthy PA and Attia ZI follow closely, also demonstrating substantial academic impact. From a temporal perspective, authors like Fukamachi K and Karimov JH have maintained consistently high productivity across multiple years, indicating their sustained contributions to artificial heart research. It is noteworthy that the research activity patterns of these core authors align closely with the overall development trend of the field; a steady increase in output is observed between 2010 and 2020, while the productivity and influence of some authors surged dramatically after 2020. This coincides with the previously analyzed global research trends, reflecting that the field's rapid development phase overlaps with the active periods of these highly productive authors.

Figure 5B clearly presents the local citation counts for individual authors. Friedman PA ranks first with 472 citations, showcasing significant academic influence. Following closely are Noseworthy PA (449 citations) and Attia ZI (383 citations), forming the top tier with the highest citation counts in the field.

### Visual analysis of journals and cited journals

**Figure 6:**
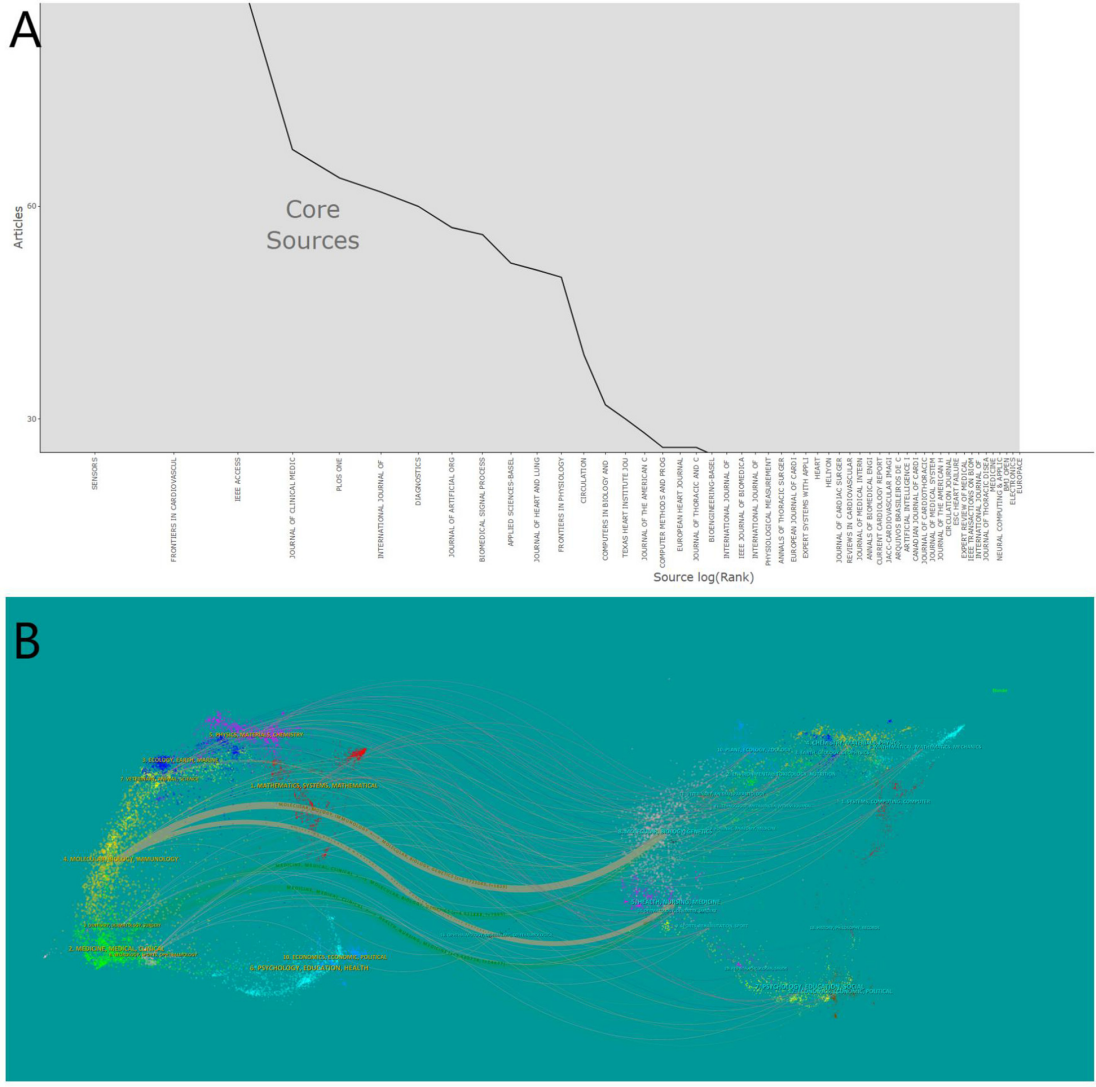
**(A)** Core sources by Bradford's Law. **(B)** Dual map coverage of journals.

Between 2010 and 2024, 1,840 journals published articles related to artificial hearts. Bradford's Law can identify the core journals within a specific field (25). As shown in Figure 6A, an analysis using Bradford's Law conducted in Bibliometrix identified 1,966 publications originating from 54 core sources, accounting for 31.16% of the total publications. Table 2 details the top 10 journals ranked by the number of publications in this research field. Web of Science provided the 2023 Journal Citation Reports (JCR) and Impact Factors (IF). The IF is a metric primarily used to evaluate the quality, importance, and influence of journals (26). Journal publication in the artificial heart research field is highly concentrated in specialized journals. Journal of Artificial Organs (296 articles), ASAIO Journal (227 articles), and Artificial Organs (176 articles) constitute the three core publishing platforms. Although these journals are highly specialized, their JCR Impact Factors are relatively moderate. Conversely, citations are dominated by high-impact general medical journals. The New England Journal of Medicine (7,565 citations, IF = 126.082), the Journal of Heart and Lung Transplantation (7,234 citations), and Circulation (6,882 citations) rank as the top three most cited journals. Top-tier medical journals such as JACC and The Lancet also receive significant citation volumes.

**Table 2:** The top 10 published journals and the top 10 co-cited journals in the field of artificial heart.

Rank	Journal	Documents	JCR	Impact factor (2024)	Co-cited journal	Citations	JCR	Impact factor (2024)
1	Artificial Organs	113	3	1.1	Circulation	6,018	1	35.5
2	ASAIO Journal	109	2	3.1	Journal of the American College of Cardiology	5,620	1	21.7
3	Scientific Reports	104	1	3.8	New England	5,292	1	96.3
4	Sensors	103	2	3.4	The Journal of Heart and Lung Transplantation	3,043	1	7.8
5	Frontiers in Cardiovascular Medicine	100	2	2.8	European Heart Journal	2,269	1	37.6
6	IEEE Access	94	2	3.4	Annals of Thoracic Surgery	2,149	2	4.6
7	Journal of Clinical Medicine	68	1	4.9	Artificial Organs	2,020	3	1.1
8	PLoS ONE	64	1	2.9	PLoS ONE	1,957	1	2.9
9	International Journal of Artificial Organs	62	4	1.33	The Journal of Thoracic and Cardiovascular Surgery	1,946	1	4.9
10	Diagnostics	60	2	2.6	ASAIO Journal	1,702	2	3.1

Figure 6B intuitively reveals the interdisciplinary nature and knowledge flow patterns within the artificial heart research field. The left side of the map aggregates the core citing journals, primarily situated in applied and engineering disciplines such as Cardiac & Cardiovascular Systems, Surgery, Transplantation, Biomedical Engineering, and Artificial Organs. This represents the main output base for research findings in the field. Conversely, the right side displays the core cited journals, concentrated in fundamental medical sciences, broader medical and clinical disciplines, foundational engineering fields like Materials Science and Fluid Dynamics, and encompassing top-tier general medical journals. This constitutes the primary source of knowledge for the field. The dense flow lines connecting the left and right sides, particularly the prominent thick lines originating from applied disciplines on the left and pointing towards basic and engineering sciences on the right, clearly indicate that artificial heart research heavily relies on and extensively draws knowledge from basic sciences, engineering principles, and authoritative clinical practices. This pronounced interdisciplinary citation pathway profoundly reflects the inherent need within the field to integrate clinical medicine, basic medical sciences, and engineering. It presents a typical knowledge ecosystem where foundational theories provide support, driving specialized technological innovation and clinical application.

### Analysis of co-cited references

Co-cited references refer to two or more documents that are cited together by other publications (27). Table 3 lists the top 10 most frequently co-cited references. The reference with the highest co-citation count is “Screening for cardiac contractile dysfunction using an artificial intelligence-enabled electrocardiogram”. This paper primarily discusses a novel method developed by researchers that utilizes artificial intelligence to analyze standard electrocardiograms, aimed at screening for asymptomatic left ventricular systolic dysfunction. By training a convolutional neural network model, this AI can accurately identify patients with impaired cardiac pumping function from routine ECG data. More importantly, the model can also predict individuals with currently normal heart function who are at a high risk of developing cardiac dysfunction in the future. This study demonstrates that AI-enabled ECG can serve as a powerful, low-cost, non-invasive, and widely accessible screening tool for the early detection and prediction of left ventricular dysfunction. Although the primary objective of AI-ECG screening is the assessment of cardiac function, its technical paradigm offers methodological references for the physiological adaptive control of artificial hearts. Therefore, it is incorporated into the analysis.

**Table 3:** Studies on artificial heart were cited as the top 10 articles.

Rank	Co-cited reference	Citations	Year	Types
1	Attia ZI, 2019, Nat Med, V25, P70, doi: 10.1038/s41591-018-0240-2	131	2019	Article
2	Hannun AY, 2019, Nat Med, V25, P65, doi: 10.1038/s41591-018-0268-3	106	2019	Article
3	Attia ZI, 2019, Lancet, V394, P861, doi: 10.1016/S0140-6736 (19)31721-0	100	2019	Article
4	Johnson KW, 2018, J Am Coll Cardiol, V71, P2668, doi: 10.1016/j.jacc.2018.03.521	83	2018	Article
5	Zhang J, 2018, Circulation, V138, P1623, doi: 10.1161/CIRCULATIONAHA.118.034338	67	2018	Review
6	Dey D, 2019, J Am Coll Cardiol, V73, P1317, doi: 10.1016/j.jacc.2018.12.054	66	2018	Review
7	Ponikowski P, 2016, Eur Heart J, V37, P2129, doi: 10.1093/eurheartj/ehw128	61	2016	Guideline
8	Benjamin EJ, 2019, Circulation, V139, PE56, doi: 10.1161/CIR.0000000000000659	56	2019	Report
9	Kirklin JK, 2015, J Heart Lung Transpl, V34, P1495, doi: 10.1016/j.healun.2015.10.003	51	2015	Report
10	Krittanawong C, 2017, J Am Coll Cardiol, V69, P2657, doi: 10.1016/j.jacc.2017.03.571	49	2017	Article

### Analysis of keywords

**Figure 7:**
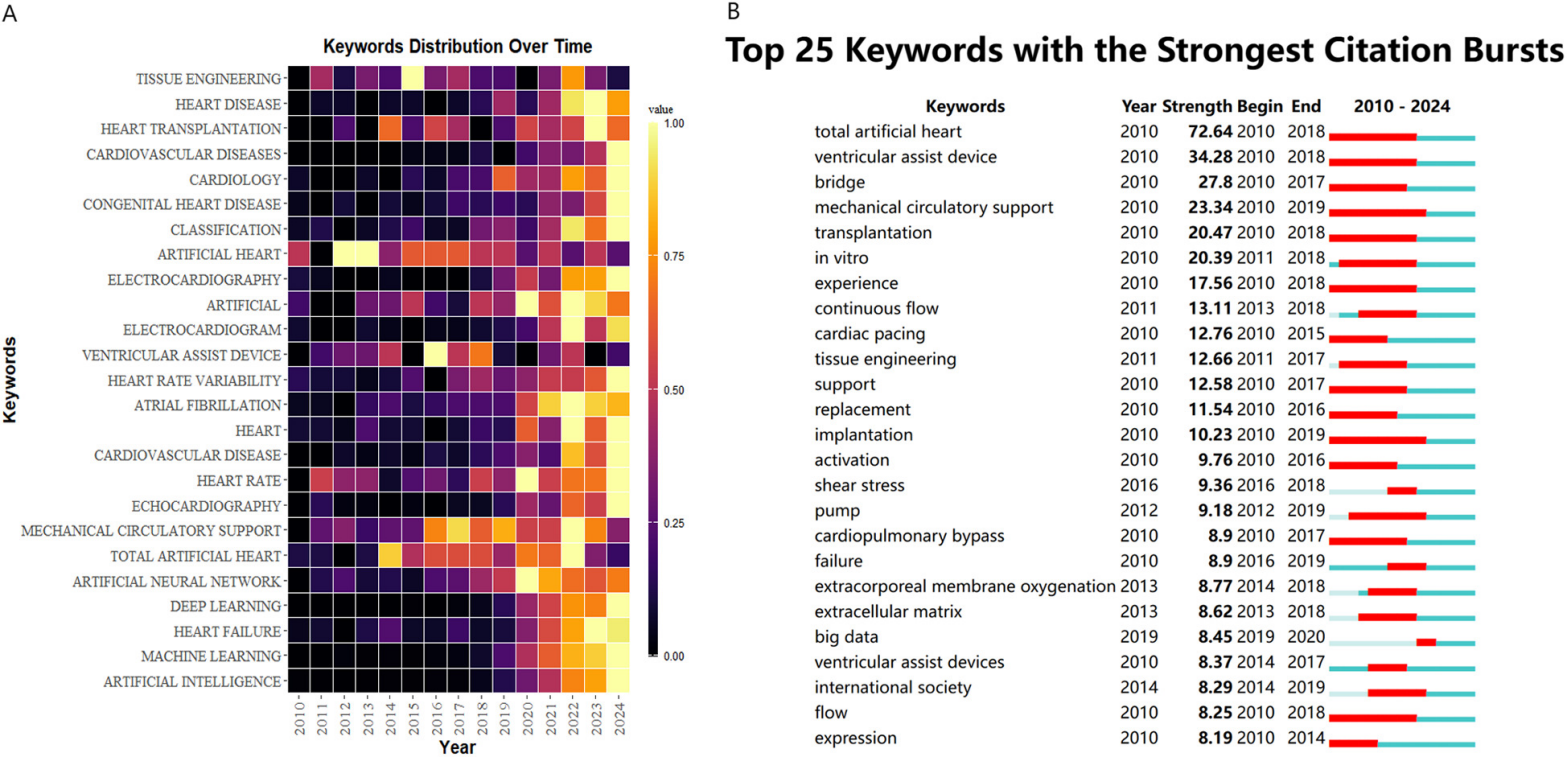
**(A)** The yearly occurrences of top 25 keywords. **(B)** Top 25 keywords with the strongest citation bursts.

Figure 7A systematically presents the disciplinary evolution pathway of the artificial heart field over 15 years (2010–2024). Visualization data show that “VENTRICULAR ASSIST DEVICE” exhibited sustained high activity levels after 2018, highly coinciding with the clinical translation period of third-generation magnetic levitation bearing technology. “TISSUE ENGINEERING” maintained high activity throughout the entire period, reflecting the core role of biomaterials in optimizing hemocompatibility. Notably, “ARTIFICIAL INTELLIGENCE” and “DEEP LEARNING” reached peak intensity values in 2023, signaling the breakthrough penetration of intelligent control algorithms into physiologically adaptive pump regulation. Meanwhile, “TOTAL ARTIFICIAL HEART” formed a local hotspot during 2020–2022, showing a temporal association with the Phase III clinical trial stage of Carmat's bioprosthetic device.

Figure 7B presents an analysis based on the keyword burst strength map from 2010 to 2024. “total artificial heart” exhibited the highest burst strength, characterizing the academic focus on this technology during the developmental stage of first-generation mechanical hearts. The concurrent bursts of “ventricular assist device” and “mechanical circulatory support” reflect the paradigm shift of ventricular assist technology towards systemic therapeutic strategies. Notably, the emergence of new keywords after 2019, such as “big data” and “continuous flow”, signals a shift in research focus towards the deep integration of intelligent hemodynamic regulation and clinical data analysis. Early basic research, represented by keywords like “cardiac pacing” and “extracellular matrix”, formed a technological iteration chain with the mid-term focus on biomechanical indicators such as “shear stress”, revealing the field's research paradigm transition from mere device development towards optimization of the bio-mechanical interface.

## Discussion

This bibliometric analysis provides a strategic roadmap for future advancements in the artificial heart field. By identifying emerging research hotspots and global collaboration patterns, it guides researchers toward underserved areas and promising interdisciplinary avenues. Furthermore, insights into the evolution of key themes, such as blood damage mitigation and regulatory challenges, can inform policy-making for device development and accelerate clinical translation by highlighting critical hurdles and successful mitigation strategies. Ultimately, this study aims to foster more targeted research investments and collaborative initiatives, thereby propelling the field toward safer, more effective, and widely accessible artificial heart technologies.

Over the period 2010–2024, the field of artificial heart research demonstrated significant and sustained growth, evidenced by an average annual increase of 13.9% in publication volume and an approximate 260-fold surge in citation counts. This remarkable expansion signals a critical transition from a phase primarily focused on technological exploration to one driven by rapid clinical application and translational impact. This observed exponential growth is not merely a quantitative increase but reflects deeper underlying mechanisms within the artificial heart field. Early growth phases were primarily driven by foundational research in biocompatible materials and fluid dynamics, essential for overcoming initial technical barriers. As these challenges were partially addressed, the field transitioned into a phase characterized by the development and clinical testing of first-generation ventricular assist devices (VADs). The surge in publications around the mid-2010s can be attributed to the maturation of long-term VAD support, crucial regulatory approvals, and increased funding for clinical trials demonstrating improved survival outcomes. Concurrently, external factors such as the increasing global prevalence of advanced heart failure and limited availability of donor hearts have created a pressing clinical demand, which in turn spurred research investment and technological innovation. Furthermore, the increasing accessibility of advanced simulation tools and *in vitro* testing platforms has significantly accelerated device design and optimization, directly contributing to this upward trend in research output. This multi-faceted interaction between clinical need, technological breakthrough, and a supportive funding and policy environment collectively explains the dynamic evolution observed in the bibliometric data. Critically, the persistent challenge of blood-device interactions, particularly the insidious effects of sublethal damage to erythrocytes and platelets under non-physiological shear stress, remains a primary driver of innovation. This nuanced understanding of blood trauma beyond overt hemolysis directly underpins the bibliometric trends towards key technologies: magnetically levitated continuous-flow pumps minimize mechanical stress, soft robotic hearts replicate gentler native hemodynamics, and AI-driven optimization enables blood-informed design of flow paths and interfaces. Thus, mitigating both lethal and sublethal blood damage is not just a challenge, it is the core impetus propelling the field's most significant advancements.

Within this rapidly evolving landscape, the United States has maintained absolute dominance, contributing 1,449 publications and garnering 40,719 citations, with its core technology R&D and clinical translation efforts spearheading industry development. While China ranked second with 925 publications, its average citation per publication was notably lower than that of the US. At the institutional level, leading North American medical institutions, such as Harvard University and the Mayo Clinic, formed robust collaborative networks leveraging extensive clinical resources and engineering expertise. Concurrently, institutions like University College London and the University of Toronto contributed unique innovations, particularly in pediatric artificial hearts and anticoagulation management. The evolution of research themes exhibited a distinct three-stage progression, reflecting these driving forces: a “Technology Foundation Phase” focused on the clinical validation of pulsatile pumps; a subsequent “Cross-Disciplinary Integration Phase” marked by an intelligent transformation, signified by keywords such as “big data” and “deep learning”; and finally, a “Precision Medicine Phase”, characterized by the emergence of research directions integrating regenerative medicine, including “personalized anticoagulation” and “biohybrid systems.”

The artificial heart field has been significantly shaped by corporate innovations. Leading companies such as Abbott (28, 29), Medtronic (30), and Carmat (31, 32) have played crucial roles in advancing device technology, materials science, and miniaturization. Their continuous investment in R&D, coupled with rigorous clinical trials, has been instrumental in extending patient survival and improving quality of life. These industry players not only drive technological progress but also foster a competitive environment that accelerates innovation in areas like pulsatile flow, biocompatibility, and transcutaneous energy transfer systems.

These technological advancements are rapidly being applied in diverse clinical scenarios with varying efficacy. In bridge-to-transplantation (BTT), devices like the HeartMate II demonstrate improved cardiac indices and enable mean support durations of 258 days pre-transplant (33), while the Heart Ware HVAD, despite requiring meticulous anticoagulation for thrombosis management, has successfully bridged hundreds of patients to transplant (34). For destination therapy (DT), the HeartMate III has shown promising median survival exceeding 18 months in advanced heart failure patients (35). In acute cardiogenic shock, strategies utilizing devices like Impella may need adjustment based on prior ECMO use (36), and adjunctive therapies can stabilize patients for mechanical support initiation (37). Pediatric populations, such as those with single ventricle physiology, rely on devices like the Berlin Heart EXCOR for survival over several months, demanding highly individualized anticoagulation to manage thrombotic and hemorrhagic risks (38). Furthermore, efficacy assessments reveal both sustained hemodynamic stability in experimental models (39) and long-term patient survival, including instances of myocardial recovery permitting device explantation. Preventive measures are evolving, such as the Jarvik 2000s retroauricular power supply design significantly reducing percutaneous lead-associated infections risk (40), and pharmacological support (41). Ongoing technological refinement focuses on magnetic levitation pumps to minimize blood trauma (42) and coatings to enhance biocompatibility (43). However, device selection, precise anticoagulation management, and comprehensive complication prevention strategies remain critical determinants of overall efficacy. Complexities arise when combining therapies, such as VAD support and VT ablation, which warrants caution regarding the risk of VT storms, especially in low EF patients (44).

While technological advancements are rapid, the clinical application and market landscape of artificial hearts also present significant challenges, particularly concerning complications and long-term management. Infection remains a major concern; for instance, percutaneous lead-associated infections rates with some HeartMate devices can reach up to 64%, significantly mitigated by design innovations like the Jarvik 2000s retro-auricular approach lowering the risk to 16% (45), yet bloodstream infections still pose urgent threats often demanding intervention within 34 days. Bleeding events are also prevalent, exemplified by gastrointestinal bleeding incidence rates around 27% (46–48). This is frequently linked to anticoagulation-related acquired von Willebrand factor deficiency and the high shear stress inherent in devices like axial flow pumps. Managing this necessitates a precarious balance between thrombotic and bleeding risks when adjusting anticoagulation strategies. The interplay between device performance and adverse effects is further highlighted by historical challenges, such as the voluntary recall of the HeartWare HVAD system by Medtronic due to potential tearing of the pump outflow graft which may cause fracture of the stress relief screw during pre-implant assembly. This underscores the critical importance of robust post-market surveillance and stringent regulatory oversight. Such events highlight the complex interplay between device design, clinical outcomes, and regulatory scrutiny. Future innovations must not only focus on performance but also ensure long-term safety, reliability, and robust post-market data collection to build greater confidence among clinicians and patients, ultimately facilitating broader adoption and clinical success.

## Conclusion

This study, through bibliometric analysis, elucidates the 15-year knowledge evolution landscape and disciplinary development trajectory within the artificial heart field. It confirms the establishment of three core research paradigms centered on intelligent regulation, biomaterial innovation, and precision medicine. Current research continues to confront three major technical challenges: optimizing biocompatibility, enhancing energy system durability, and managing long-term anticoagulation. Future research must endeavor to construct integrated bio-hybrid systems founded upon a “materials-device-algorithm” framework, thereby facilitating the transition of artificial hearts from bridge-to-transplantation therapy towards physiologically adaptive, permanent replacement solutions. Concurrently, leveraging global collaborative networks is essential to accelerate the translation of fundamental research findings into clinical practice, ultimately aiming to achieve revolutionary breakthroughs in the therapy for advanced heart failure.

### Limitations

Several limitations of this study should be acknowledged. First, the analysis was restricted to data from the Web of Science Core Collection (WoSCC) to ensure software compatibility, potentially excluding relevant studies indexed in other databases such as PubMed, Google Scholar, and Embase. Second, emerging high-quality publications may not have received sufficient representation due to lower citation frequencies characteristic of recently published works. Finally, the exclusive inclusion of English-language literature may introduce selection bias, particularly regarding region-specific research contributions. These constraints should inform cautious interpretation of the findings while highlighting opportunities for methodological refinement in future bibliometric investigations.
