# Supplementary material for: Mapping the research landscape of artificial hearts: identifying hotspots and frontiers through bibliometric analysis
Source: Front Cardiovasc Med. 2025 Sep 30;12:1613605. doi: 10.3389/fcvm.2025.1613605 (PMC12518402; doi:10.3389/fcvm.2025.1613605)
Supplement: Supplementary file 1 [file Datasheet1.pdf]

Entry Terms:

Artificial Heart

Artificial Hearts

Hearts, Artificial

Web of Science Search Strategy (v0.1)

Database: Web of Science Core Collection

Entitlements:

- WOS.IC: 1993 to 2025
- WOS.CCR: 1985 to 2025
- WOS.SCI: 1900 to 2025
- WOS.AHCI: 1975 to 2025
- WOS.ESCI: 2020 to 2025
- WOS.ISTP: 1996 to 2025
- WOS.SSCI: 1900 to 2025
- WOS.ISSHP: 1996 to 2025

Searches:

1: ((TS=(Artificial Heart)) OR TS=(Artificial Hearts)) OR TS=(Hearts, Artificial) Editions: WOS.SCI Date Run: Mon Jun 02 2025 23:53:29 GMT+0800 (中国标准时间) Results: 12917

2: ((TS=(Artificial Heart)) OR TS=(Artificial Hearts)) OR TS=(Hearts, Artificial) and 2024 or 2023 or 2022 or 2021 or 2020 or 2019 or 2018 or 2017 or 2016 or 2015 or 2014 or 2013 or 2012 or 2011 or 2010 (Publication Years) Editions: WOS.SCI Date Run: Mon Jun 02 2025 23:53:50 GMT+0800 (中国标准时间) Results: 7349

3: ((TS=(Artificial Heart)) OR TS=(Artificial Hearts)) OR TS=(Hearts, Artificial) and 2024 or 2023 or 2022 or 2021 or 2020 or 2019 or 2018 or 2017 or 2016 or 2015 or 2014 or 2013 or 2012 or 2011 or 2010 (Publication Years) and Meeting Abstract or Editorial Material or Proceeding Paper (Exclude – Document Types) Editions: WOS.SCI Date Run: Mon Jun 02 2025 23:54:01 GMT+0800 (中国标准时间) Results: 6631

4: ((TS=(Artificial Heart)) OR TS=(Artificial Hearts)) OR TS=(Hearts, Artificial) and 2024 or 2023 or 2022 or 2021 or 2020 or 2019 or 2018 or 2017 or 2016 or 2015 or 2014 or 2013 or 2012 or 2011 or 2010 (Publication Years) and Meeting Abstract or Editorial Material or Proceeding Paper

(Exclude – Document Types) and Early Access or Letter or Retracted Publication (Exclude – Document Types) Editions: WOS.SCI Date Run: Mon Jun 02 2025 23:54:04 GMT+0800 (中国标准时间) Results: 6486

5: ((TS=(Artificial Heart)) OR TS=(Artificial Hearts)) OR TS=(Hearts, Artificial) and 2024 or 2023 or 2022 or 2021 or 2020 or 2019 or 2018 or 2017 or 2016 or 2015 or 2014 or 2013 or 2012 or 2011 or 2010 (Publication Years) and Meeting Abstract or Editorial Material or Proceeding Paper (Exclude – Document Types) and Early Access or Letter or Retracted Publication (Exclude – Document Types) and Book Chapters or Book Review or Correction (Exclude – Document Types) Editions: WOS.SCI Date Run: Mon Jun 02 2025 23:54:08 GMT+0800 (中国标准时间) Results: 6455

6: ((TS=(Artificial Heart)) OR TS=(Artificial Hearts)) OR TS=(Hearts, Artificial) and 2024 or 2023 or 2022 or 2021 or 2020 or 2019 or 2018 or 2017 or 2016 or 2015 or 2014 or 2013 or 2012 or 2011 or 2010 (Publication Years) and Meeting Abstract or Editorial Material or Proceeding Paper (Exclude – Document Types) and Early Access or Letter or Retracted Publication (Exclude – Document Types) and Book Chapters or Book Review or Correction (Exclude – Document Types) and News Item or Biographical-Item or Bibliography (Exclude – Document Types) Editions: WOS.SCI Date Run: Mon Jun 02 2025 23:54:13 GMT+0800 (中国标准时间) Results: 6443

7: ((TS=(Artificial Heart)) OR TS=(Artificial Hearts)) OR TS=(Hearts, Artificial) and 2024 or 2023 or 2022 or 2021 or 2020 or 2019 or 2018 or 2017 or 2016 or 2015 or 2014 or 2013 or 2012 or 2011 or 2010 (Publication Years) and Meeting Abstract or Editorial Material or Proceeding Paper (Exclude – Document Types) and Early Access or Letter or Retracted Publication (Exclude – Document Types) and Book Chapters or Book Review or Correction (Exclude – Document Types) and News Item or Biographical-Item or Bibliography (Exclude – Document Types) and Meeting or Reprint or Retraction (Exclude – Document Types) Editions: WOS.SCI Date Run: Mon Jun 02 2025 23:54:17 GMT+0800 (中国标准时间) Results: 6413

8: ((TS=(Artificial Heart)) OR TS=(Artificial Hearts)) OR TS=(Hearts, Artificial) and 2024 or 2023 or 2022 or 2021 or 2020 or 2019 or 2018 or 2017 or 2016 or 2015 or 2014 or 2013 or 2012 or 2011 or 2010 (Publication Years) and Meeting Abstract or Editorial Material or Proceeding Paper (Exclude – Document Types) and Early Access or Letter or Retracted Publication (Exclude – Document Types) and Book Chapters or Book Review or Correction (Exclude – Document Types) and News Item or Biographical-Item or Bibliography (Exclude – Document Types) and Meeting or Reprint or Retraction (Exclude – Document Types) and English (Languages) Editions: WOS.SCI Date Run: Mon Jun 02 2025 23:54:23 GMT+0800 (中国标准时间) Results: 6310
